# Negative depletion mediated brightfield circulating tumour cell identification strategy on microparticle-based microfluidic chip

**DOI:** 10.1186/s12951-020-00623-4

**Published:** 2020-05-07

**Authors:** Shuibing Wang, Shaoli Hong, Shijia Cai, Jia Lei, Jinyao Chen, Nangang Zhang, Zhao Ai, Kan Liu, Man Tang

**Affiliations:** 1 School of Electronic and Electrical Engineering, Wuhan Textile University, Wuhan, 430200 People’s Republic of China; 2 Hubei Engineering and Technology Research Center for Functional Fiber Fabrication and Testing, Wuhan Textile University, Wuhan, 430200 People’s Republic of China; 3 Hubei Province Engineering Research Center for Intelligent Micro-nano Medical Equipment and Key Technologies, Wuhan, 30200 People’s Republic of China

**Keywords:** Circulating tumour cell, Negative depletion, Microfluidic chip, Brightfield, Identification

## Abstract

**Background:**

The most convenient circulating tumor cells (CTCs) identification method is direct analysis of cells under bright field microscopy by which CTCs can be comprehensive studied based on morphology, phenotype or even cellular function. However, universal cell markers and a standard tumour cell map do not exist, thus limiting the clinical application of CTCs.

**Results:**

This paper focuses on an automatic and convenient negative depletion strategy for circulating tumour cell identification under bright field microscopy. In this strategy, immune microparticles (IMPs) are applied to negatively label white blood cells rather than the tumour cells, such that tumour cells can be directly distinguished under brightfield of the microscopy. In this way, all of the heterogeneous tumour cells and their phenotype properties can be retained for further cancer-related studies. In addition, a wedge-shaped microfluidic chip is constructed for heterogeneous CTC pre-purification and enrichment by size, thus significantly decreasing the interference of haematological cells. Additionally, all cell treatments are processed automatically, and the tumour cells can be rapidly counted and distinguished via customized cell analytical software, showing high detection efficiency and automation. This IMPs based negative cell labelling strategy can also be combined with other classic cell identification methods, thus demonstrating its excellent compatibility.

**Conclusion:**

This identification strategy features simple and harmless for tumour cells, as well as excellent accuracy and efficiency. And the low equipment demand and high automation level make it promise for extensive application in basic medical institutions.

## Background

As a new kind of biomarker for cancer, detection of circulating tumour cells (CTCs) may pave the way towards non-invasive cancer diagnosis in the clinic [1, 2]. Many reports have shown that a change in the CTC quantity may significantly reveal cancer progress [3, 4]. However, CTC identification is one of the most difficult problems that need to be solved. First, processes for cell identification may damage cell viability or morphology [5, 6]. For example, the RT-PCR analyses must destroy the cell to extract its nucleic acid, and immunofluorescent involves the challenging problems of cell fixation and permeation, and the heterogeneity of tumour cells. Both methods damage the cell’s original characteristics. In fact, certain more comprehensive and in-depth studies on cancer must keep the captured CTCs intact or even alive [7, 8]. Of most importance, expensive instruments such as fluorescence inverted microscope or confocal microscope are necessary, but basic medical institutions cannot afford these instruments [9]. Additionally, due to the inherent heterogeneity of CTCs, no universal labels exist that can be used in CTC identification [10].

Hence, a convenient and universal method is urgently needed for CTC identification. It is well known that, the most convenient cell identification method is direct analysis of cells under brightfield microscopy. The cell morphology or phenotype can be easily distinguished, and the equipment required for detection is notably convenient. In the absence of a standard tumour cell map, CTCs need to be further verified with brightfield microscopy. Recently, researchers have devoted efforts to labelling tumour cells with multiplex fluorescence markers [11–14], but the inherent heterogeneity and absence of universal markers make it difficult to collect and identify all of the CTCs from blood. Certain special CTCs might also be omitted in this way. On the other side, the cell morphology or phenotype characteristics are destroyed after traditional immunofluorescence identification. Fluorescence photobleaching and spectral overlap also limit long term and multi-biomarkers cell identification [15].

CTCs exist in the bloodstream with the feature of rarity [16, 17]. Specifically, the complex and large number of haematological cells in blood may cover up special biomarkers in blood, which can influence the diagnostic result and efficiency [18–20]. Thus, pre-purification is necessary. The physical properties of CTCs are distinct relative to those of blood cells, e.g., cell size, shape, and deformability [21, 22]. By taking advantage of the similar size of cells, the microfluidic chip has made great contributions to CTC enrichment and purification [23]. With the aid of the microfluidic chip, CTCs can be separated from blood cells by many size-based methods, such as micro-filtration [24, 25], hydrodynamic chromatography [26, 27], dielectrophoretic methods [28, 29]. Compared with antigen recognition techniques, the microfluidic chip-based physical property distinction method can effectively collect heterogeneous CTCs from blood, even those that have lost epithelial markers when undergoing epithelial-to-mesenchymal transitions [30, 31].

**Scheme 1:**
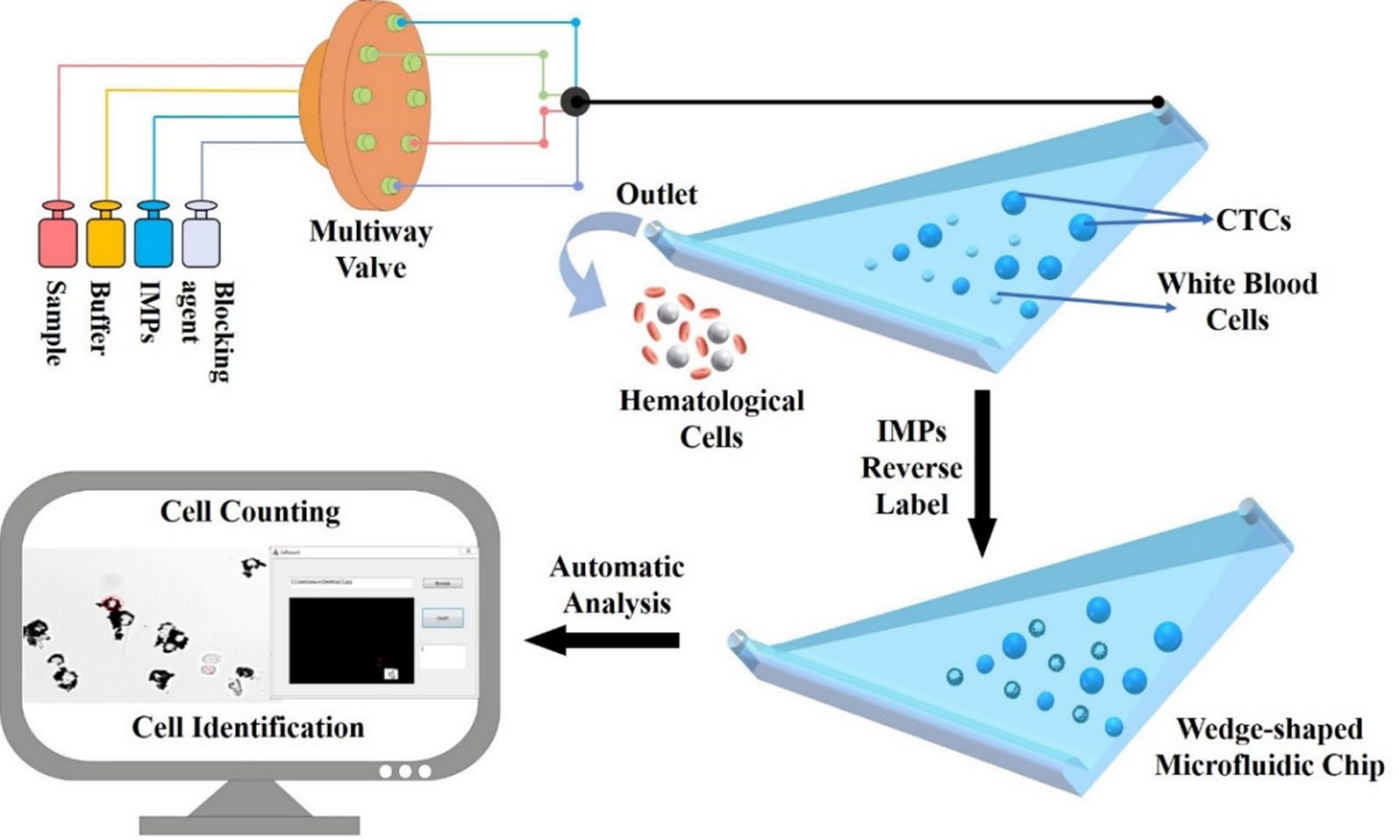
Scheme of the proposed circulating tumour cell automatic identification strategy based on a wedge-shaped microfluidic chip and cell negative depletion by IMPs

In our previous work, a wedge-shaped microfluidic chip was fabricated for CTC detection and clinical application [32, 33]. This chip achieved CTC enrichment from different types of solid tumours via a “label-free strategy”, and the clinical significance of CTCs in gastric cancer was studied. Herein, a new microfluidic chip-based strategy is proposed for CTC detection and identification (Scheme 1). In this strategy, a wedge-shaped microfluidic chip is first used to enrich the CTCs from blood cells. Then, instead of fluorescence labelling of CTCs, immune microparticles (IMPs, modified with anti-CD45 antibody) are applied to exclude the retained cells that are highly similar to the morphology of the CTCs in the chip, and thus CTCs are identified in a reverse manner. In this way, CTCs can be identified and analysed under brightfield microscopy, which effectively avoids the overlap of different fluorescent labels. The entire strategy is simple, without any harmful steps, and other immunocytochemistry or staining methods for CTC identification can also be combined with this method. It is worth mentioning that, all of the processes can be automated, even identification. Since the IMPs can be observed under brightfield microscopy, fluorescence modules are not necessary for the microscope, which simplifies and reduces the cost. This strategy combines the advantages of bright field identification and negative immune cell recognition and proposes a new strategy for further CTC-related studies.

## Results and discussion

### Characterization of the wedge-shaped microfluidic chip

**Fig. 1:**
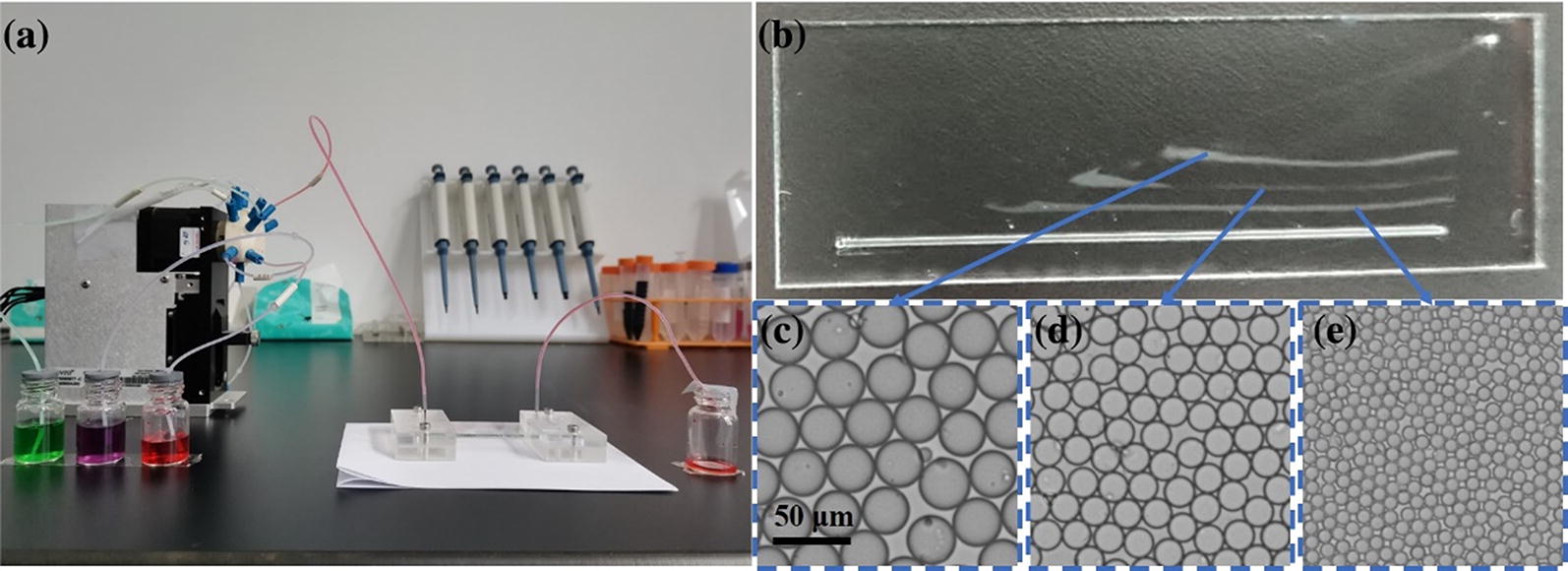
**a** Photograph of the integrated automatic cell negative depletion equipment for tumor cell pre-purification. The left side shows a multiway valve, and the right side depicts the wedge-shaped microfluidic chip. The three bottles with different colors are sample bottles for the cell sample, PBS and IMPs, respectively. **b** Characterization of the microfluidic chip by microspheres with different sizes. **c** Photograph of 30 μm microparticles in the first line. **d** Photograph of 20 μm microparticles in the second line. (3) Photograph of 10 μm microparticles in the third line

It is well known that the size and stiffness of tumour cells are larger than those of WBCs. Based on this observation, WBCs and RBCs can be excluded prior to CTC identification, and thus the interference of blood cells can be reduced. In this work, a wedge-shaped microfluidic chip was applied to exclude most of the blood cells and the CTCs could be subsequently identified by cell morphology in a first step. Compared with the existing micro-filtration chip method, this wedge-shaped microfluidic chip could effectively separate and enrich CTCs directly from the whole blood without clogging. As shown in Additional file 1: Figure S1A, the wedge-shaped chip maximum height was 60 μm, and its minimum height was 5 μm. Cells with size up to 5 μm, stiff nucleus and hard deformation such as CTCs are trapped in the chip while blood cells are washed-out. Hence, CTCs could be separated and purified from the blood before further identification. The cut-off height of this wedge-shaped chip could also be lower than 5 μm. As the CTCs were separated from the blood cells by size and stiffness, CTCs with different phenotypes could be collected and identified in the chip. A multiway valve was applied for sample introduction and washing, thus reducing manual restrictions and enhancing automation (Fig. 1a).

To better demonstrate the internal structure of the wedge-shaped microfluidic chip, microparticles with different sizes (10 μm, 20 μm, and 30 μm) were simultaneously introduced into the chip. As Fig. 1b–e showed, microparticles with a size of 30 μm were captured first, and the 20 μm and 10 μm microparticles were captured sequentially. Microparticles with different sizes could be separated at fixed locations without any overlap. It was also worth mentioning that, the spaces between the location of the 10 μm microparticle and 20 μm microparticle and the distance between the 20 μm microparticle and 30 μm microparticle were nearly the same, which exhibited the uniformity slope and displayed the exquisite design of this wedge-shaped microfluidic chip.

**Fig. 2:**
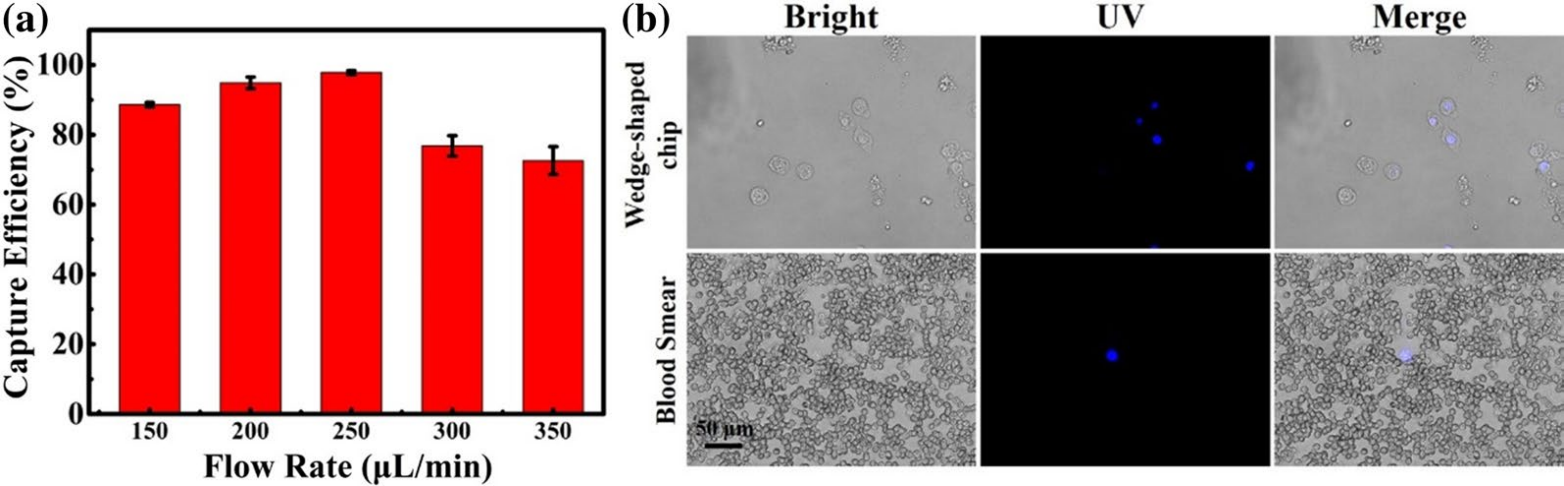
**a** Relationship of the flow rate and tumor cell capture efficiency. Error bars represent the standard deviations of triplicate experiments. **b** MCF-7 cell distribution in wedge-shaped chip and blood smear

Breast cancer MCF-7 cells were applied as a model to investigate the cell separation efficiency of the wedge-shaped microfluidic chip. The flow rates used to introduce MCF-7 cells into the chip were set as 150, 200, 250, 300, and 350 μL/min, and their cell capture efficiencies were measured in terms of the ratio of the number of captured cells *versus* the total number of captured and uncaptured cells. From Fig. 2a, the capture efficiencies for MCF-7 were increased as the flow rate increased from 150 μL/min to 250 μL/min, and the tumour cell capture efficiencies decreased sharply after the flow rate increased up to 250 μL/min. This result could be explained as the large liquid pressure that was caused by the high flow rate and might result in cell deformation or even disruption, thus causing the cell to break away from the chip. According to this observation, the flow rate for cell separation was optimized at 250 μL/min. Additionally, as shown in Additional file 1: Figure S2, the cell morphologies of MCF-7 cells in the wedge-shaped microfluidic chip were similar to those on the glass slide, showing the capability of tumour cell morphological analysis in the chip.

In addition to cell separation, this wedge-shaped microfluidic chip could also purify tumour cells from the whole blood. To simulate blood samples from cancer patients, approximately 100 nuclear-stained MCF-7 cells were spiked into 1 mL blood. As shown in Fig. 2b, the fluorescence signal from Hoechst 33,342 could be minimally observed from the blood sample, even when cells were already tiled as a monolayer. Tumor cells were enriched and purified in the wedge-shaped microfluidic chip with few white blood cells and nearly no red blood cells. Moreover, about 150 liver tumour cells Hep 3b cells, Bel 7402 cells, and BT 747 cells and breast tumour cells SK-BR-3 cells were spiked into 2 mL blood as a simulated clinical sample, and all of these tumour cells with different phenotypes can be captured in the chip with high efficiency (Additional file 1: Figure S3). Hence, heterogeneous CTCs were supposed to be easily purified or enriched by this wedge-shaped chip without overlap, and they can also be identified under brightfield microscopy without haematological cell interference. In this way, tumour cell could be rapidly identified by cell morphology in the wedge-shaped chip firstly.

### Labelling capability of the IMPs on white blood cells

**Fig. 3:**
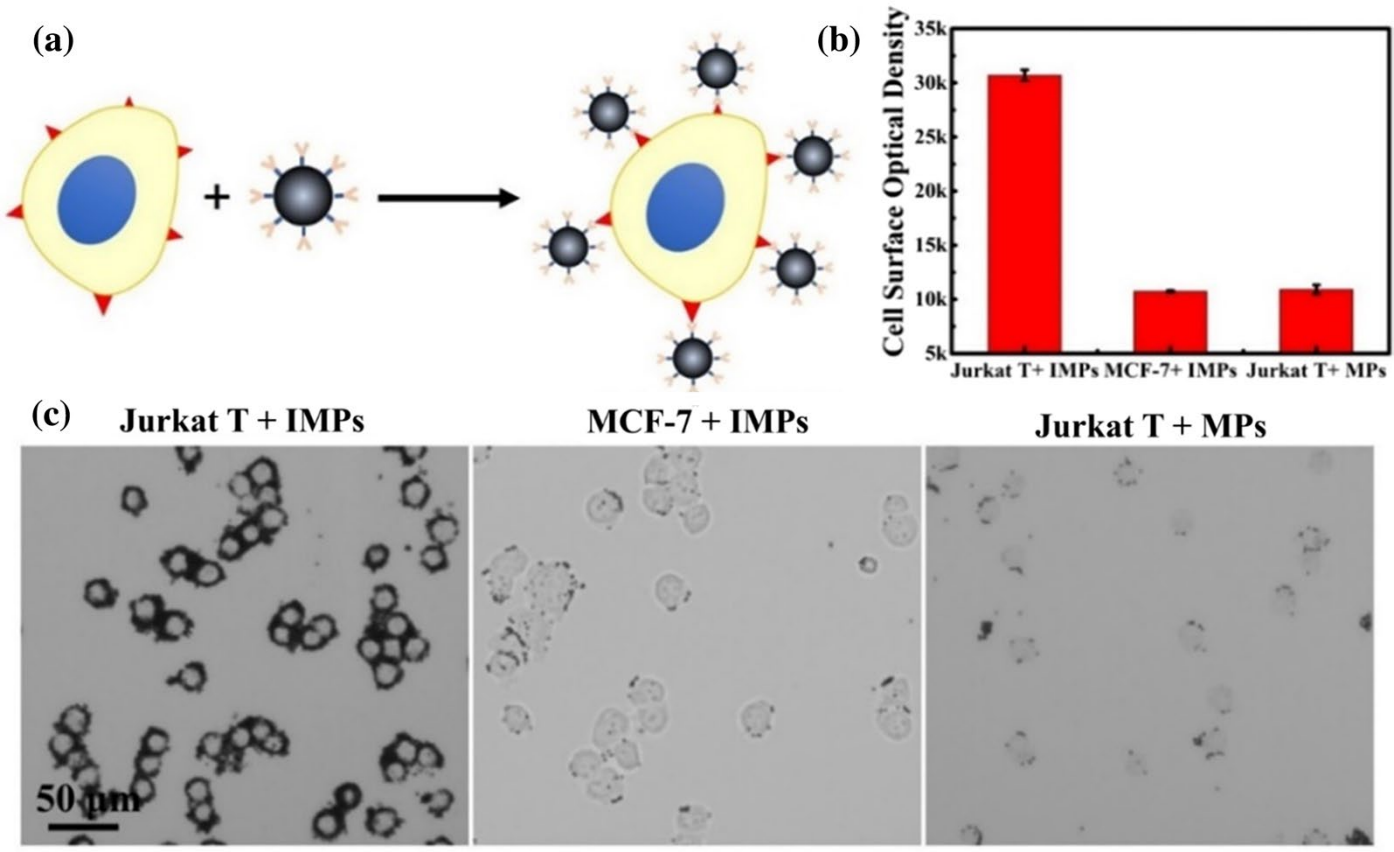
**a** Schematic diagram for the process of IMPs labelling with MCF-7 cells. **b** Efficiency and specificity of cells (MCF-7 cells and Jurkat T cells) labelled by IMPs and MPs, respectively. Error bars represent the standard deviations of triplicate experiments. **c** Bright images of Jurkat T cells labelled by IMPs, MCF-7 cells labelled by IMPs, and Jurkat T cells labelled by MPs, respectively

The labelling capability of the immune microparticles (IMPs) on white blood cells was investigated. Jurkat T cells, a type of human blood leukemia cells, were used as model. Breast tumour cell MCF-7 cells were also applied for comparison. The microparticles (MPs) were successfully modified with anti-CD 45 antibody prior to each experiment (Additional file 1: Figure S4). In this section, all of the microparticles with similar size could be applied in this negative depletion strategy after conjugation with the corresponding antibody. The microparticles should also be observed with a normal optical microscope. To approve the concept of the labelling capability of the IMPs on white cells, mixed cells (MCF-7 cells and Jurkat T cells) were introduced into the desired wedge-shaped separation chip for enrichment and purification first. The IMPs were then applied to label the retained Jurkat T cells in the microfluidic chip for negative depletion (Fig. 3a). To optimize the labelling efficiency, the process of IMP introduction was repeated three times with opposite direction. As shown in Fig. 3b, c, the surface of the Jurkat T cells could be successfully labelled by abundant IMPs, and hardly any IMPs reacted with MCF-7 cells. MPs without antibody modification were minimally labelled to Jurkat T cells as well. The cell surface optical density statistics further confirmed these results. All of the above evidence underscored the high specificity and efficiency of this white blood cell negative depletion strategy. Specifically, as Fig. 3c shows, most of the unlabeled MCF-7 cells retained their original morphology, and their phenotype expression also remained, which laid the foundation for the further CTC identification using brightfield microscopy.

**Fig. 4:**
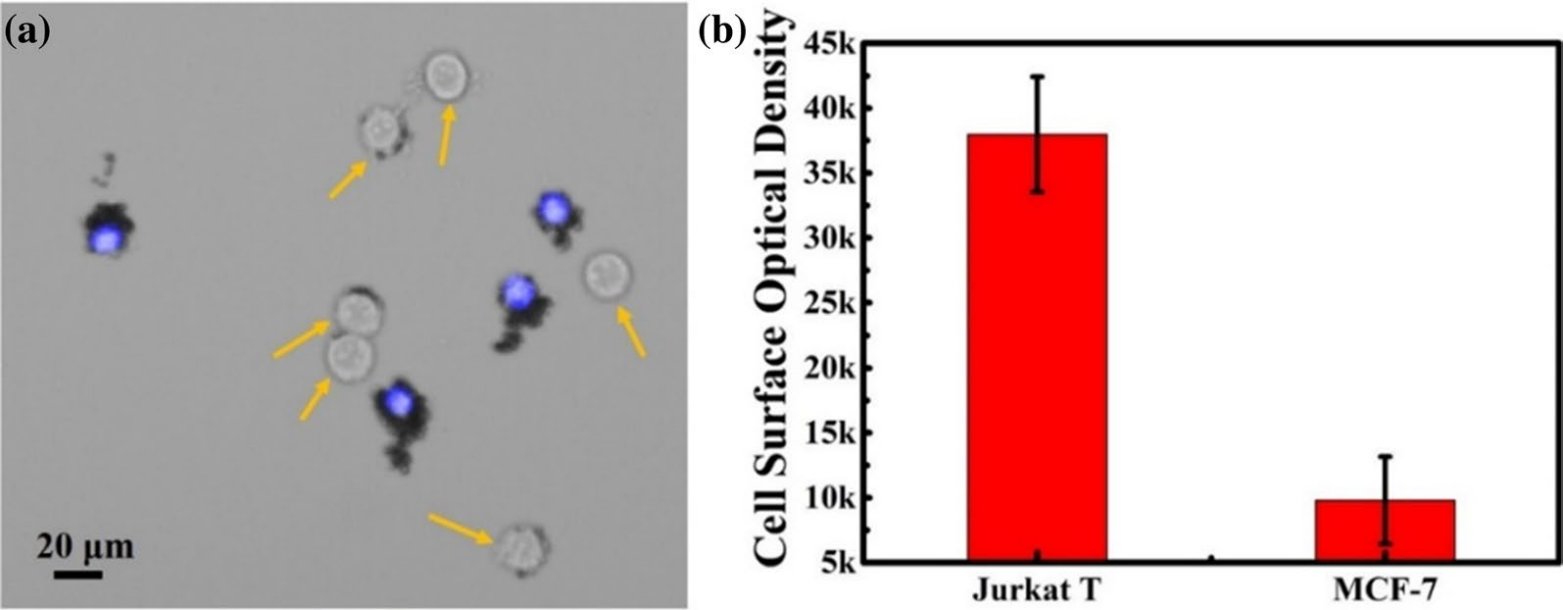
**a** Images of IMPs labelled on mixed cells (Jurkat T cells and MCF-7 cells). Arrows point to the MCF-7 cells that were not nuclear-stained prior to experiments. **b** Label efficiency of IMPs towards mixed cells containing MCF-7 cells and Jurkat T cells (proportions of 1:1). Error bars represent the standard deviations of triplicate experiments

Microparticles could be used to efficiently label the normal cells and distinguish the CTCs from background. Hence, the ability of the IMPs to label the WBCs from background was investigated. As a function of the wedge-shaped microfluidic chip, approximately 99.87% to 99.95% of white blood cells could be eluted, and the number of tumour cells was low [32]. To keep the number of MCF-7 cells and Jurkat T cells consistent, a cell mixture containing 10^4^ MCF-7 cells and 10^4^ Jurkat T cells was applied, and a wedge-shaped chip was fabricated with a 4 μm minimum height. Approximately 90.4 ± 2.2% Jurkat T cells and 94.9 ± 1.7% MCF-7 cells were captured in the chip. For the same protocol, IMPs could efficiently label the surface of the Jurkat T cells but minimally reacted with the MCF-7 cells (Fig. 4). As a result, the conclusion could be drawn that this method was capable of specifically labelling Jurkat T cells from a high background. More importantly, due to the colour of the IMPs, an obvious difference between the Jurkat T cells and MCF-7 cells could be directly observed under a brightfield microscope. Compared with the existing methods, this strategy of labelling the white blood cells instead of tumour cells did not require any tumour-related biomarkers and the cells retained their original morphotype (Table 1).

**Table 1:** Summary of CTC identification methods

Method	Material demands	Target	Equipment	Refs.
Fluorescence	Fluorescent or near-infrared fluorescent	Tumour cells	Fluorescent or near-infrared fluorescent module	[11, 14, 34]
Electrochemical sensor	Electrochemical signal	Tumour cells	Electrochemical workstation	[29, 35]
Magnetic label	Magnetism	Tumour cells	Magnetic assay reader	[36, 37]
Negative depletion method	Unlimited	WBCs	Bright module	

### Compatibility of the identification method

**Fig. 5:**
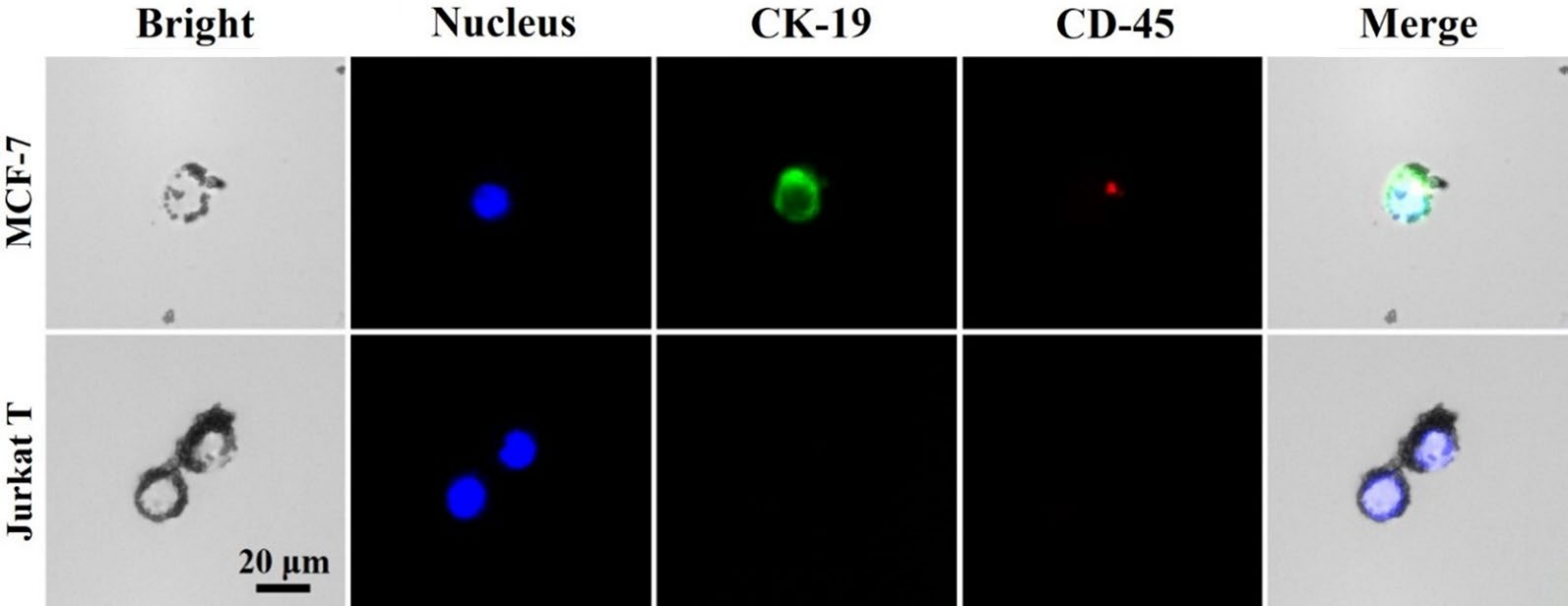
Images of IMPs labelled on the mixed cells (Jurkat T cells and MCF-7 cells), and the results of the immunocytochemistry identification method

To further confirm this identification method, a commonly used immunocytochemistry identification (ICC identification) method was applied after IMPs based cell identification. A marker for epithelial cells known as Cytokeratin 19 (CK-19) and a kind of leukocyte common antigen known as CD 45 were applied for identification. As shown in Fig. 5, MCF-7 cells that were not labelled by IMPs had a high level of CK-19 antigen expression and had no CD 45 antigen expression. For Jurkat T cells labelled by IMPs, neither CK-19 nor CD-45 antigen was found. These results showed that the results of the proposed cell identification method were in accordance with the commonly used immunocytochemistry identification results. Thereinto, the loss of CD-45 expression in Jurkat cells might be due to active site occupation by IMPs. The MCF-7 cells could be labelled with the anti-CK 19 antibody, showing that morphotype and phenotype were faultlessly retained.

On the other hand, according to the result of Fig. 4, the range of each cell mean density (cell surface integrated optical density versus cell superficial area) were defined in three components: cells with a mean density less than 20 can be considered as tumour cells, cells with a mean density up to 30 of mean density are considered as white blood cells, and cells with a mean density range of 20 to 30 are considered as suspected tumour cells. Then, cells identified by immunocytochemistry from three repeated experiments were randomly chosen for statistical analysis. As Additional file 1: Table S1 showed, approximately 93.3 ± 3.3% of cells could be correctly identified as tumour cells and approximately 87.5 ± 5.3% of cells could be correctly identified as normal cells using our proposed negative depletion method. The successful ICC identification after the IMPs labelling also revealed the compatibility of the proposed CTC bright field identification strategy, which might extend itself to multi-model identification of tumour cells. For better clinical application, Liu’s staining method, which was widely used in blood cell morphology and phenotype analysis in pathology, was also attempted. Hence, this method has a high potential for popularization in clinical applications. Moreover, homemade software was designed for automatic analysis of the results of cell identification using this strategy (Additional file 1: Figure S6). Thus, a comprehensive and automatically tumour identification separation and identification strategy was successfully constructed, which might pave the way for a new clinical point-of-care test.

## Conclusion

This paper proposes a new strategy for direct tumor cell identification under brightfield microscopy. The integrated strategy has several distinctive advantages: (1) Tumour cells are effectively separated and enriched from the blood through a wedge-shaped microfluidic chip. Thus, all of the red blood cells and almost all of the white blood cells can be excluded. Additionally, all of the heterogeneous tumour cells can be retained. (2) IMPs are applied to label the retained white blood cell in the wedge-shaped chip in a reverse manner, thus retaining the original characteristics of the tumour cells for further comprehensive research. Tumor cells can be identified and analysed directly under brightfield microscopy, and hence this strategy can be realized using commonly used microscopy with no fluorescent modules. Compared with the existing cell identification method, which requires a variety of expensive labellers, this method drastically reduces the cost for tumour diagnosis. (3) The IMPs based tumour cell identification method can also be used in combination with other cell identification methods, such as immunocytochemistry identification and Liu’s staining. (4) For better ease of use, all processes in this strategy, which contain cell pre-purification, cell labelling and cell identification analysis, can be automated, thus greatly improving the cell identification efficiency. In summary, this automatic and convenient cell negative depletion and identification strategy is highly promising for extensively application in basic medical institutions.

## Materials and methods

### Materials

Microparticle (SEQ-PC-02, 1.0–2.0 μm, 50 mg/10 mL) were purchased from Tianjin Saierqun Technology Co. Ltd. Microparticles (20 µm, 20 µm, 30 µm) were also bought from Suzhou Knowledge & Benefit Sphere Tech. Co., Ltd. EDC (*N*-(3-dimethylaminopropyl)-*N*′-ethylcarbodiimide hydrochloride), NHS (*N*-hydroxysuccinimide), BSA (bovine serum albumin) and anti-EpCAM monoclonal antibody were purchased from Sigma-Aldrich. FITC labeled anti-Cytokeratin 19 (anti-CK19) monoclonal antibodies and allophycocyanin (APC) labeled anti-CD45 monoclonal antibodies were obtained from Abcam.

Breast cancer MCF-7 cells, and Jurkat T cells (human peripheral blood leukaemia T cells) were purchased from the China Type Culture Collection. All media for cell culture were purchased from Gibco Corp, Biosharp and ExCell Biology Inc. Ultrapure water (18 MΩ cm) was processed by a HITECH laboratory water purification system.

Fluorescence images were recorded by a charge-coupled device (CCD) camera (QImaging optiMOS) mounted on an inverted fluorescence microscope (Olympus IX83). Syringe pump was sourced from Longer Precision Pump Co., Ltd. Microchannels in the microfluidic device were constructed by a computerized numerical control (CNC) engraving and milling machine (JDLGC16_A8, Jingdiao Group Co., Ltd.).

### Cell culture

MCF-7 cells, Hep 3b cells, Bel 7402 cells, BT 747 cells and SK-BR-3 cells were cultured in cell culture flasks at 37 °C in a humidified atmosphere with 5% CO_2_ (in air) with DMEM. supply. The DMEM supplement contained 10% foetal bovine serum and 100 IU/mL penicillin–streptomycin. After detachment by trypsin, cells were centrifuged at 1500 rpm for 5 min. Finally, the precipitated cells were transferred into PBS for use.

Jurkat T cells were cultured in common cell culture flasks at 37 °C in a humidified atmosphere with 5% CO_2_ (in air) 37 °C with 1640 supply. The 1640 supplement contained 10% foetal bovine serum and 100 IU/mL penicillin–streptomycin. Finally, cells were centrifuged at 900 rpm for 5 min and then precipitated cells were transferred into PBS for use. All cells were purchased from the China Type Culture Collection. The blood samples were kindly provided by healthy volunteers.

Cell staining was processed by Hoechst 33,342 (10 μg/mL) for 30 min and washed by centrifugation at 900 rpm for 5 min prior to the experiments. The stained cells were transferred into PBS for use.

### Fabrication of wedge-shaped microfluidic chip

The wedged-shaped microfluidic chip was built in our previous work. The fabrication method applied the wet etching technique and thermal bonding technique. The entire structure of this chip consisted of a wedge-shaped microchamber and a linear reservoir. A stepping motor equipped with the wet etching technique was applied for fabrication of the wedge-shaped microchamber. The maximum and minimum height of the wedge-shaped microfluidic chip were designed as 60 μm and 4 or 5 μm. The linear reservoir was made using a computerized numerical control (CNC) engraving instrument, and its height and width were approximately 500 μm and 200 μm, respectively.

### Tumour cell separation and labelling

Hoechst 33,342-stained Jurkat T cells and unstained MCF-7 cells were introduced into the wedge-shaped microfluidic chip at a flow rate of 200 μm/min. PBS was subsequently applied to wash the microchamber and remove the uncaptured haematological cells. Anti-CD45 antibody-modified immune microparticles (IMPs) were loaded into the chip three times repeatedly. The extra IMPs were excluded by PBS for several minutes. The cells could be identified in the bright field under the microscope. The cell capture efficiency was calculated by the ratio of the number of captured cells versus the total number of captured and uncaptured cells. The statistics for the cell surface optical density were calculated as the IOD sum versus the number of cells. The IOD sum was obtained by Image Pro Plus software. The homemade software could also be used in direct and automatic identification of the tumour cells from white blood cells.

Immunocytochemistry identification was processed directly in the chip after IMPs labelling. Labelled cells were fixed with 4% paraformaldehyde (20 min, 2 μL/min) and permeabilized with 0.1% Triton-X 100 (20 min, 2 μL/min) first. A commonly used immunocytochemistry identification protocol consisting of 30 μg/mL DAPI, FITC-labelled anti-CK19 monoclonal antibodies and APC-labelled anti-CD45 monoclonal antibodies was applied to the chip (30 min, 1 μL/min). After washing by PBS, the cell phenotype and morphology could be identified under inverted fluorescence microscopy.

Liu’s staining is improved from the Romanowsky staining method using the Liu A stain of Eosin Y to dye the cytoplasm as red and the Liu B stain of Azur I to dye the cell nucleus as blue. Liu’s staining is processed by introducing Liu A stain into the chip for seconds, followed by a mixture of Liu A stain and Liu B stain (1:2). After reaction for 2 min, PBS was introduced for washing. The cells could subsequently be identified under a microscope.

## Supplementary information

**Additional file 1: Figure S1.** (A) Schematic diagram of microfluidic chip for tumour cell pre-purification, with detailed structural parameters labelled. Left side: top view of the microfluidic chip, right side: lateral view of the microfluidic chip. (B) Photograph of the integrated automatic cell negative depletion equipment for tumor cell pre-purification. The right side shows a multiway valve and the left side depicts the wedge-shaped microfluidic chip. **Figure S2.** Characterization of the morphology of MCF-7 cells in the wedge-shaped microfluidic chip and on a glass slide. **Figure S3.** Capture efficiencies of the wedge-shaped chip towards Hep 3b cells, Bel 7402 cells, BT 747 cells and SK-BR-3 cells. **Figure S4.** (A) Schematic diagram for modification of MPCs with anti-CD 45 antibody. (B) Bright image, fluorescence image and their merged image of the IPCs (up row) and MPs (bottom row) after reaction with Alex 488-labelled second antibody. **Figure S5.** (A) Basic procedures in the designed homemade automatic software. (B) Output result of automatic cell identification. (C) Basic interface of the homemade automatic software. **Table S1.** Statistical data from immunocytochemistry identification.

## Data Availability

All data generated or analysed during this study are included in this article and its additional file.
