# Supplementary material for: Negative depletion mediated brightfield circulating tumour cell identification strategy on microparticle-based microfluidic chip
Source: J Nanobiotechnology. 2020 May 7;18:70. doi: 10.1186/s12951-020-00623-4 (PMC7206695; doi:10.1186/s12951-020-00623-4)
Supplement: Supplementary file 1 — Additional file 1: Figure S1. (A) Schematic diagram of microfluidic chip for tumour cell pre-purification, with detailed structural parameters labelled. Left side: top view of the microfluidic chip, right side: lateral view of the microfluidic chip. (B) Photograph of the integrated automatic cell negative depletion equipment for tumor cell pre-purification. The right side shows a multiway valve and the left side depicts the wedge-shaped microfluidic chip. Figure S2. Characterization of the morphology of MCF-7 cells in the wedge-shaped microfluidic chip and on a glass slide. Figure S3. Capture efficiencies of the wedge-shaped chip towards Hep 3b cells, Bel 7402 cells, BT 747 cells and SK-BR-3 cells. Figure S4. (A) Schematic diagram for modification of MPCs with anti-CD 45 antibody. (B) Bright image, fluorescence image and their merged image of the IPCs (up row) and MPs (bottom row) after reaction with Alex 488-labelled second antibody. Figure S5. (A) Basic procedures in the designed homemade automatic software. (B) Output result of automatic cell identification. (C) Basic interface of the homemade automatic software. Table S1. Statistical data from immunocytochemistry identification. [file 12951_2020_623_MOESM1_ESM.pdf]

Negative Depletion Mediated Brightfield Circulating Tumour Cell Identification Strategy on Microparticle-Based Microfluidic Chip

Shuibing Wang [†a, b, c], *Shaoli Hong,* [† a, b, c] *Shijia Cai* [a], *Jia Lei* [a], *Jinyao Chen* [a], *Nangang Zhang,* [a, b, c] *Zhao Ai,* [a, b, c] *Kan Liu* [*a, b, c], *and Man Tang* [*a, b, c]

[a] School of Electronic and Electrical Engineering, Wuhan Textile University, Wuhan 430200, People's Republic of China.

[b] Hubei Engineering and Technology Research Center for Functional Fiber Fabrication and Testing, Wuhan Textile University, Wuhan 430200, People's Republic of China.

[c] Hubei Province Engineering Research Center for Intelligent Micro-nano Medical Equipment and Key Technologies, Wuhan 30200, People's Republic of China

[*] Co-corresponding e-mail: mtang@wtu.edu.cn; (M. Tang); liukan2002@gmail.com; (K. Liu);

[†] These authors contributed equally to this work.

S.1. Characterization of the wedge-shaped microfluidic chip

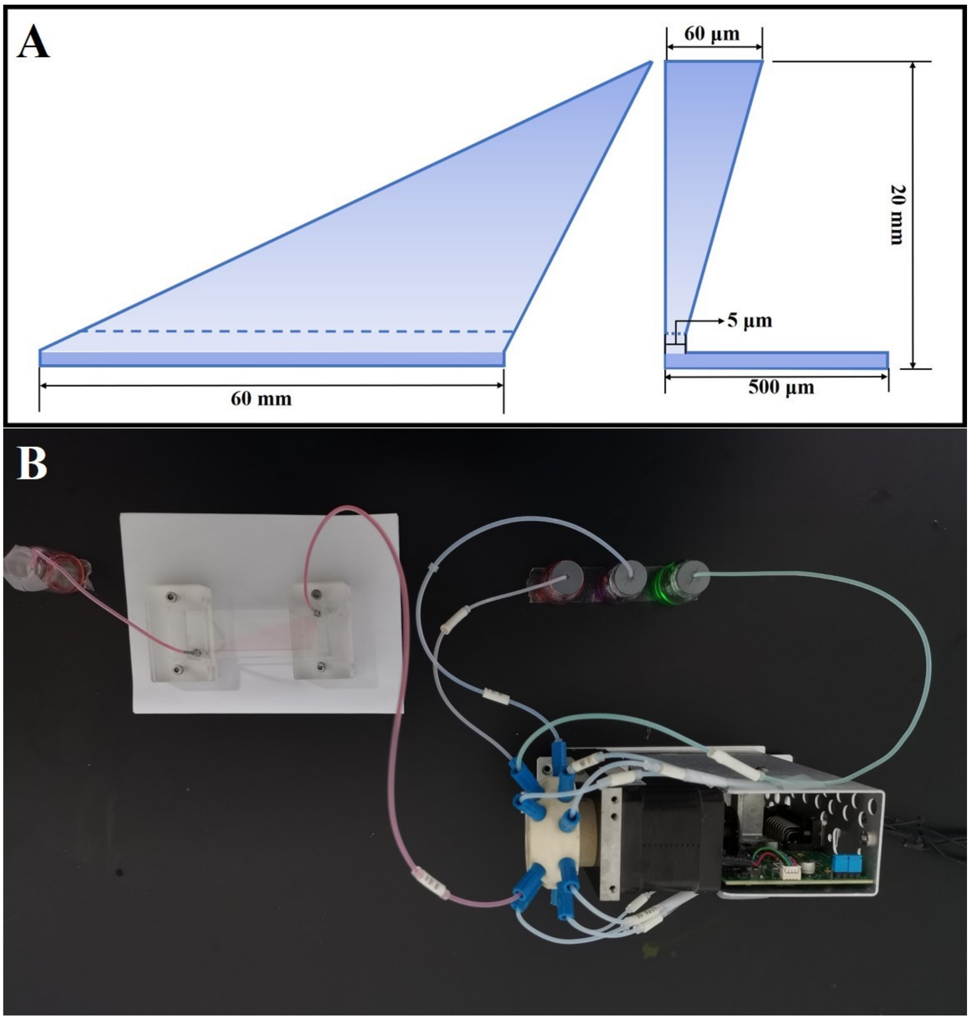

Figure S1. (A) Schematic diagram of a microfluidic chip for tumour cell pre-purification, with detailed structural parameters labelled. Left side: top view of the microfluidic chip, right side: lateral view of the microfluidic chip. (B) Photograph of the integrated automatic cell negative depletion equipment for tumour cell pre-purification. The right side shows a multiway valve and the left side depicts the wedge-shaped microfluidic chip.

S.2. Characterization the morphology of MCF-7 cells.

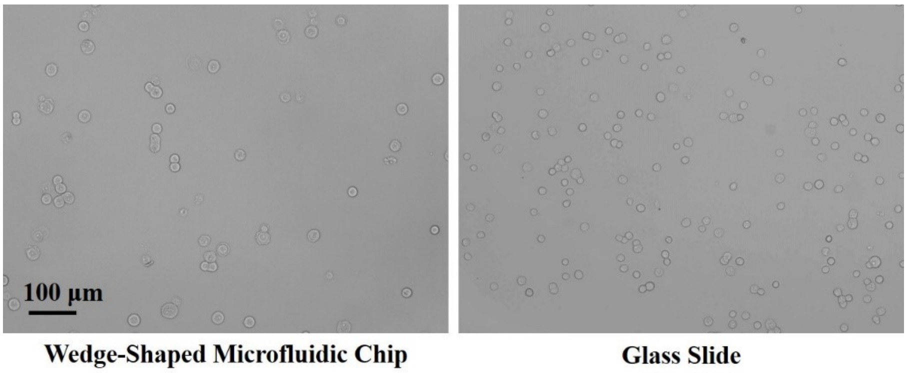

Figure S2. Characterization of the morphology of MCF-7 cells in the wedge-shaped microfluidic chip and on a glass slide.

S.3. Characterization of the cell capture ability to different tumour cells.

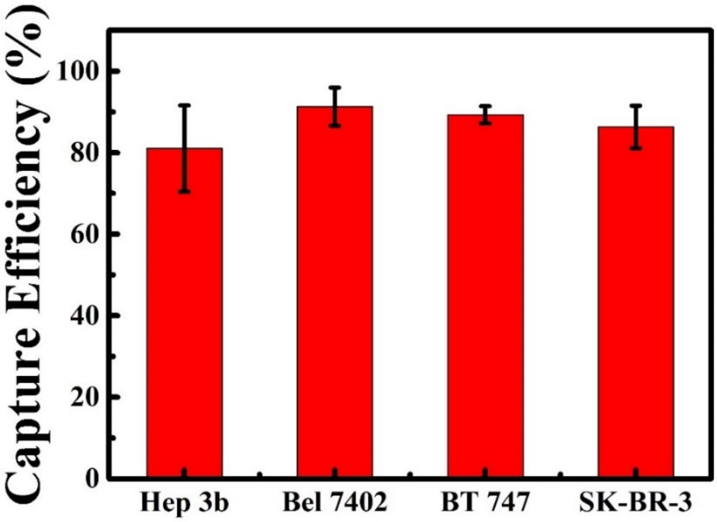

Figure S3. Capture efficiencies of the wedge-shaped chip towards Hep 3b cells, Bel 7402 cells, BT 747 cells and SK-BR-3 cells.

S.4. Characterization of the IMPs

The surface of the immune microparticles (MPs) was the carboxyl, so the antibody could be modified through the reaction between the carboxyl and the amino group. In this work, for labelling the white blood cells in the blood, an anti-CD45 antibody was applied for immune microparticles modification (IMPs). The CD45 antigen is a leukocyte common antigen which was high expression on the most of white blood cells. To investigate the success conjugation between MPs and anti-CD45 antibody, Alex 488 labelled second antibody was used to react with the obtained IMPs. From the fluorescence microscopy images in **Figure S4B**, almost all the IMPs could be marked with green light, while no green light could be observed in MPs. These results indicated the successful modification of anti-CD45 antibody on the MPs and the modified antibody still kept active for the following experiments.

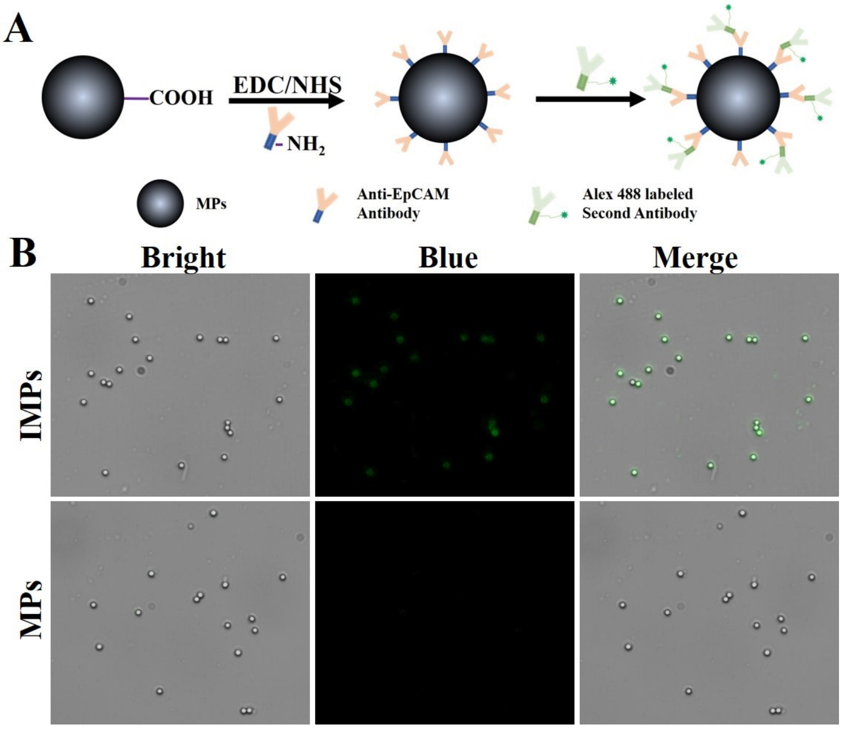

Figure S4. (A) Schematic diagram for modification of MPs with anti-CD 45 antibody. (B) Bright image, fluorescence image and their merged image of the IMPs (up row) and MPs (bottom row) after reaction with Alex 488-labelled second antibody.

S.5. Confirming of the result of the negative depletion strategy.

According to the result of **Figure 4** in manuscript, the range of each cell mean density (cell surface integrated optical density *versus* cell superficial area) were defined in three components: cells with a mean density less than 20 can be considered as tumour cells, cells with a mean density up to 30 are considered as white blood cells, and cells with a mean density range of 20 to 30 are considered as suspected tumour cells. About 90 tumour cells and 240 Jurkat T cells (30 tumour cells and 80 Jurkat T cells for each experiment) identified by immunocytochemistry from three repeated experiments were used for statistic. As **Table S1** showed, approximately 93.3±3.3 % of cells could be correctly identified as tumour cells and approximately 87.5±5.3 % of cells could be correctly identified as normal cells using our proposed negative depletion method.

Table S1. Statistical data of the negative depletion method.

**Range of the Cell Mean Density**	**Recovery Rate of MCF-7 cells (%)**	**Recovery Rate of Jurkat T cells (%)**
< 20	93.3±3.3	3.1±2.6
>20 and <30	5.6±1.9	9.4±7.9
>30	1.11±1.9	87.5±5.3

S.6.Homemade software for cell identification

In the proposed cell identification strategy, tumour cell could be reversely revealed from the white blood cells by the IMP label. But the processes for cell counting and identification were still tedious and time-consuming, so a homemade software was designed. As **Figure S5A** showed, this designed software concluding shape recognition, cell surface optical density statistics, size screening, and cell number counting. After sequence images were taken, this software could directly count the unlabelled tumour cells in seconds. As it exhibited in **Figure S5B-C**, tumour cell could be marked and count automatically. Moreover, a manual mode was added for calibration and validation. Based on these above, a comprehensive and automatically tumour identification separation and identification strategy was constructed, that hope to pave a new way for the clinical point-of-care test.

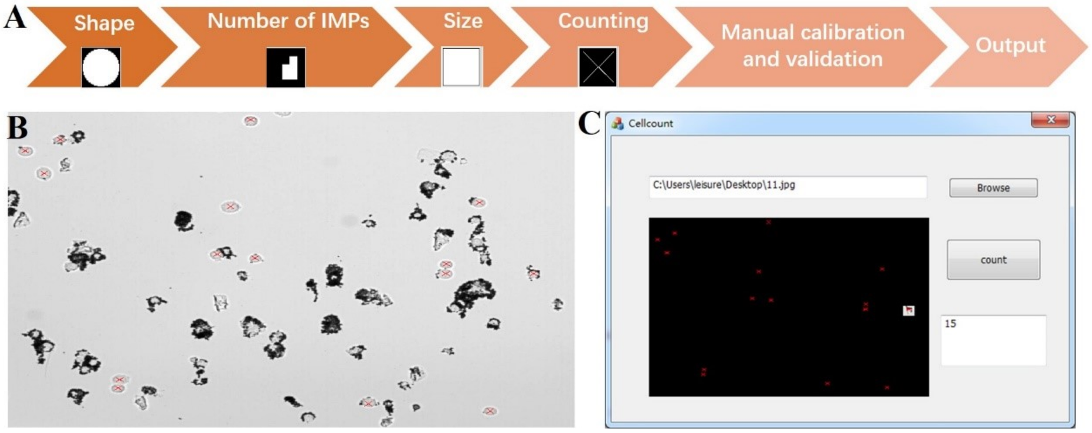

Figure S5. (A) Basic procedures in the designed homemade software. (B) Output result of automatic cell identification. (C) Basic interface of the homemade automatic software.
